# KIF22 promotes multiple myeloma progression by regulating the CDC25C/CDK1/cyclinB1 pathway

**DOI:** 10.1007/s00432-024-05747-w

**Published:** 2024-05-07

**Authors:** Meng Zhai, Jiyu Miao, Ru Zhang, Rui Liu, Fangmei Li, Ying Shen, Ting Wang, Xuezhu Xu, Gongzhizi Gao, Jinsong Hu, Aili He, Ju Bai

**Affiliations:** 1 Department of Hematology, The Second Affiliated Hospital of Xi’an Jiaotong University, Xi’an, China; 2 Xi’an Key Laboratory of Hematological Diseases, Xi’an, China; 3 National Local Joint Engineering Research Center of Biodiagnostics and Biotherapy, The Second Affiliated Hospital of Xi’an Jiaotong University, Xi’an, China; 4 Department of Tumor and Immunology in Precision Medical Institute, Xi’an Jiaotong University, Xi’an, China; 5 Department of Cell Biology and Genetics, Xi’an Jiaotong University Health Science Center, 76 Yanta West Road, Xi’anShaanxi, 710061 China

**Keywords:** Multiple myeloma, KIF22, CDC25C, Proliferation, Cell cycle

## Abstract

**Purpose:**

Multiple myeloma (MM) is an incurable hematological malignancy characterized by clonal proliferation of malignant plasma B cells in bone marrow, and its pathogenesis remains unknown. The aim of this study was to determine the role of kinesin family member 22 (KIF22) in MM and elucidate its molecular mechanism.

**Methods:**

The expression of KIF22 was detected in MM patients based upon the public datasets and clinical samples. Then, in vitro assays were performed to investigate the biological function of KIF22 in MM cell lines, and subcutaneous xenograft models in nude mice were conducted in vivo. Chromatin immunoprecipitation (ChIP) and luciferase reporter assay were used to determine the mechanism of KIF22-mediated regulation.

**Results:**

The results demonstrated that the expression of KIF22 in MM patients was associated with several clinical features, including gender (*P* = 0.016), LDH (*P* < 0.001), β_2_-MG (*P* = 0.003), percentage of tumor cells (BM) (*P* = 0.002) and poor prognosis (*P* < 0.0001). Furthermore, changing the expression of KIF22 mainly influenced the cell proliferation in vitro and tumor growth in vivo, and caused G2/M phase cell cycle dysfunction. Mechanically, KIF22 directly transcriptionally regulated cell division cycle 25C (CDC25C) by binding its promoter and indirectly influenced CDC25C expression by regulating the ERK pathway. KIF22 also regulated CDC25C/CDK1/cyclinB1 pathway.

**Conclusion:**

KIF22 could promote cell proliferation and cell cycle progression by transcriptionally regulating CDC25C and its downstream CDC25C/CDK1/cyclinB1 pathway to facilitate MM progression, which might be a potential therapeutic target in MM.

**Supplementary Information:**

The online version contains supplementary material available at 10.1007/s00432-024-05747-w.

## Introduction

Multiple myeloma (MM), the second most common hematological malignancy, is an incurable clonal plasma cell dysplasia, which mainly occurs in the elderly population (Liu et al. 2019). In past decades, with the emerging novel agents and laboratory methods, the diagnosis and treatment of MM have been significantly improved. Especially, the therapy of MM ranges from traditional chemotherapy to proteasome inhibitors (PIs)-based triple-drug combination treatment, and immunotherapy including monoclonal antibodies (mAbs) and chimeric antigen receptor-engineered T (CAR-T) cells therapy have achieved great clinical response in some MM patients (Rajkumar and Kumar 2020). However, relapsed and refractory MM  and drug resistance still remain great clinical challenges due to the unclear molecular mechanism and heterogeneity of MM patients.

Kinesin superfamily proteins (KIFs) are microtubule-dependent molecular motor proteins that transport organelles, protein complexes, mRNAs and chromosomes in a microtubule- and ATP-dependent manner (Hirokawa et al. 2015; Miki et al. 2005). Up to now, there are more than 45 members of KIFs family in mammalian cells. KIF22, also known as kinesin-like DNA-binding protein (Kid), is a member of the kinesin-10 family. KIF22 is involved in the regulation of microtubule stability, synaptic development, and cytoskeletal dynamics, especially during mitosis (Tokai et al. 1996). Studies have revealed that KIF22 was overexpressed in many cancers, such as lung cancer (Pike et al. 2018), gastric cancer (Yu et al. 2020), breast cancer (Yu et al. 2014), and prostate cancer (Zhang et al. 2018), and was associated with poor prognosis. Mechanically, KIF22 was reported to promote proliferation and migration in gastric cancer by regulating the MAPK–ERK pathway. In prostate cancer, the high expression of KIF22 was involved in tumor progression and adverse clinical outcome (Zhang et al. 2018).

The cell division cycle 25 (CDC25) family proteins include three isoforms, CDC25A, CDC25B, and CDC25C in mammalian cells. CDC25 family proteins can activate the complexes of cyclin-dependent kinase (CDK) with corresponding cyclin chaperones, which in turn regulate cell cycle progression (Boutros et al. 2007). These proteins are also pivotal components of the cell cycle checkpoint pathways that regulate cell division and DNA damage response. During the cell cycle, CDC25C activates the cyclin B1/CDK1 complex by triggering the dephosphorylation of CDK1, which facilitates the G2/M transition of mitotic cells (Liu et al. 2020). Down-regulation of CDC25C can induce cell cycle arrest at the G2/M phase, and its abnormal expression is associated with cancer initiation, development, metastasis and poor prognosis (Ozen and Ittmann 2005; Ying et al. 2018). It has been reported that CDC25C could be regulated by ERK1/2, which regulates its activation during G2/M transition in mammalian cells (Eymin et al. 2006).

However, studies have revealed that KIF22 is overexpressed in many cancers and associated with poor prognosis. The role of KIF22 and its underlying mechanism in MM remain unclear. In this study, we detected the expression and analyzed the clinical characteristics of KIF22 based on the Gene Expression Omnibus (GEO) database, The Cancer Genome Atlas (TCGA) dataset, and MM patients. Then, we silenced and overexpressed the expression of KIF22 in vivo and in vitro to investigate the biological function in MM. Finally, we explored the underlying mechanisms of KIF22 in transcriptionally regulating CDC25C and its downstream CDK1/cyclinB1. The findings indicated that high expression of KIF22 was associated with a higher tumor burden in MM and related with poor prognosis.

## Materials and methods

### MM patients

Bone marrow samples were obtained using aspiration from 51 MM patients and 21 healthy donors. All MM patients were diagnosed according to the International Myeloma Working Group (IMWG). The study has been approved by the Ethics Committee of the Second Affiliated Hospital of Xi'an Jiaotong University (No. 2015186). All patients provided written informed consent. The general clinical characteristics and laboratory data of the 51 MM patients are summarized in Table 1.

**Table 1:** Association between KIF22 expression and clinicopathological characteristics of the 51 patients with multiple myeloma

Clinical characteristics	Types	Number (*n* = 51)	KIF22 expression Mean ± SD		*P* value
Gender	male	26		7.705 ± 1.298	**0.016**
	female	25		6.860 ± 1.225	
Age (years)	< 65	37		7.335 ± 1.226	0.584
	≥ 65	14		7.113 ± 1.427	
LDH (IU/L)	< 250	27		6.669 ± 1.422	** < 0.001**
	≥ 250	24		7.955 ± 0.572	
β_2_-MG (mg/L)	< 5.5	21		7.866 ± 0.987	**0.003**
	≥ 5.5	30		6.859 ± 1.301	
ALB (g/L)	< 35	21		7.106 ± 1.540	0.466
	≥ 35	30		7.392 ± 1.062	
Hb (g/L)	< 100	32		7.182 ± 1.419	0.509
	≥ 100	19		7.429 ± 0.999	
Scr (umol/L)	< 177	42		7.154 ± 1.323	0.148
	≥ 177	9		7.834 ± 0.860	
BUN (mmol/L)	< 7.5	36		7.210 ± 1.329	0.582
	≥ 7.5	15		7.428 ± 1.158	
Type	IgG	20		7.267 ± 1.421	0.488
	IgA	5		6.649 ± 1.350	
	IgD	6		7.508 ± 1.298	
	Light chain	19		7.481 ± 1.020	
	NSMM	1		–	
D-S stage	I	1		–	0.597
	II	2		7.095 ± 0.318	
	III	48		7.267 ± 1.308	
ISS stage	I	4		7.464 ± 0.455	0.050
	II	13		6.387 ± 1.658	
	III	34		7.591 ± 1.011	
R-ISS stage	I	3		7.293 ± 0.368	0.789
	II	26		7.202 ± 1.388	
	III	22		7.356 ± 1.242	
mSMART	standard risk	13		7.186 ± 1.485	0.775
	high risk	38		7.304 ± 1.214	
Percentage of tumor cells (BM)	< 40%	14		6.421 ± 1.403	**0.002**
	≥ 40%	37		7.596 ± 1.074	
Cytogenetic abnormality	del RB1	23			
	Amp 1q21	25			
	del (17p)	2			
	del 13q14	12			
	t (11;14)	3			
	t (4;14)	2			
	t (14;16)	2			
	t (6;14)	1			
	Other IGH rearrangements	15			

*Hb* hemoglobin; *ALB* albumin; *Scr* serum creatinine; *LDH* lactate dehydrogenase; *β*_*2*_*-MG* β_2_-microglobulin; *BUN* blood urea nitrogen; *NSMM* non-secretory MM *Del* delete; *Amp* amplify; *IGH* immunoglobulin heavy chain genes

Bold values indicates statistically significant p values less than 0.05

### Bioinformatics and survival analysis

RNA sequence data of 15 healthy donors and 73 newly diagnosed multiple myeloma patients from the GEO dataset GSE6477 were selected to analyze the expression of KIF22 in MM. The overall survival data of 858 MM patients were collected from the TCGA GDC MMRF-COMMPASS database, and the event-free survival data of 65 MM patients were collected from the GSE4452 dataset. MM patients were divided into two groups according to appropriate gene expression cutoff values. Kaplan–Meier survival analysis was used to calculate the survival rates of the two groups. ROC curves based on KIF22 expression in MM patients and healthy donors were plotted.

### Cell lines and reagents

The human MM cell lines RPMI 8226 and MM.1S were purchased from Cell Bank, Chinese Academy of Sciences, and OPM2 was provided by the Department of Cell Biology and Genetics, Xi'an Jiaotong University. The mycoplasma testing was negative. MM cells were maintained in RPMI 1640 (HyClone; GE Healthcare Life Sciences) with 10% fetal bovine serum (FBS, Biological Industries, Kibbutz Beit Haemek, Israel), penicillin (10,000 U/L, BioSharp, Hefei, China), and streptomycin (100 mg/L, BioSharp, Hefei, China). All cells were incubated at 37 °C in a humidified atmosphere with 5% CO_2_. The CDC25C inhibitor NSC95397 (Cat#HY-108543, 10 mM × 1 mL in DMSO) was purchased from MedChemExpress (New Jersey, NJ, USA).

### Transfection of small interfering RNA (siRNA)

According to the manufacturer's instruction, MM cells were incubated with RFect^SP^siRNA/miRNA Transfection Reagent (BIOG-11026, BaiDai biology) and siRNA for 20 min and then MM cells were seeded in 12-well plates at a density of 3 × 10^5 ^ cells and cultured with complete medium without antibiotics in an incubator at 37 °C with a 5% CO_2_ atmosphere for 24–72 h. The siRNAs were synthesized by GenePharma (Shanghai, China) and the target sequences of siRNA are as follows:

negative control (siNC): forward, 5′-UUCUCCGAACGUGUCACGUTT-3′,

                         reverse, 5′-ACGUGACACGUUCGGAGAATT-3′.

siKIF22#1: forward, 5′- AGACGCUUCUACCUAGACA-3′,

                   reverse, 5′- UGUCUAGGUAGAAGCGUCU -3′.

siKIF22#2: forward, 5′- CAAGGGCCUUCGGCUAAAA -3′,

                   reverse, 5′- UUUUAGCCGAAGGCCCUUG -3′.

siKIF22#3: forward, 5′- CAGUCGAAAUCGGACUGUA-3′,

                   reverse, 5′- UACAGUCCGAUUUCGACUG-3′.

### Lentiviral transfection

The LV-shKIF22 lentiviral vector (GeneChem Co., Ltd., Shanghai, China) was transfected into RPMI 8226 cells to construct the stable KIF22 knockdown cell line. The OE-KIF22 lentiviral vector (GeneChem Co., Ltd., Shanghai, China) was transfected into RPMI 8226 and OPM2 cells to construct the stable KIF22 overexpression cell lines. All lentivirus-transfected cells were checked for transfection efficiency by observing green fluorescent protein (GFP) under an inverted fluorescence microscope (IX73; Olympus, Tokyo, Japan), reverse transcription-quantitative (RT-q) PCR and Western blotting. The cells were screened with 2 ug/mL puromycin for at least 3 weeks to construct stable expression cell lines.

### RNA isolation and reverse transcription-quantitative (RT-q) PCR

Total RNA was extracted from bone marrow mononuclear cell (BMSCs) samples of MM patients, healthy donors, and MM cells using TRIzol® reagent (Invitrogen; Thermo Fisher Scientific, Inc.). RNA purity and concentration were detected by NanoDrop ND-1000 (Thermo Fisher Scientific, Inc.). cDNA was synthesized by PrimerScrift Reverse Transcriptase (Takara Biotechnology Co., Ltd.) according to manufacturer's instruction. Then, RT-qPCR was performed by SYBR Premix Ex Taq™ II (TliRNaseH Plus; Takara Biotechnology Co., Ltd.) and a CFX96 Touch™ Real-Time PCR Detection System (Bio-Rad Laboratories, Inc.). The primer sequence of KIF22, CDC25A, CDC25B, CDC25C, and GAPDH were as follows:

KIF22: forward, 5′- AGAGATTGCTAACTGGAGGAACC-3′,

 reverse, 5′- ACCTGCATAGATGTCCTGCTG -3′.

CDC25C: forward, 5′- ATGACAATGGAAACTTGGTGGAC -3′,

 reverse, 5′- GGAGCGATATAGGCCACTTCTG -3′.

CDC25B: forward, 5′- GCATGGAGAGTCTCATTAGTGC -3′,

 reverse, 5′- CTCCGCCTCCGCTTATTCT -3′.

CDC25A: forward, 5′- GTGAAGGCGCTATTTGGCG -3′,

 reverse, 5′- TGGTTGCTCATAATCACTGCC -3′.

GAPDH: forward, 5′- CGGAGTCAACGGATTTGGTCGTAT -3′,

 reverse, 5′- AGCCTTCTCCATGGTGGTGAAGAC -3′.

### Western blotting

Total protein was isolated from MM cells and mononuclear cells collected from bone marrow blood of three newly diagnosed MM patients and three healthy normal donors selected randomly, using RIPA buffer with protease and phosphatase inhibitor cocktail (P002, New Cell and Molecular Biotech Co., LTD, China). Protein concentration was detected by the BCA assay (PP0102, Xianfeng Biotechnology Co., LTD, China). Cell debris was removed by centrifugation (15 min, 12,000 rpm), and then the sample buffer was added. An equivalent amount of protein from cell extracts was separated by a 10% SDS-PAGE and then transferred onto 0.45 μm polyvinylidene fluoride (PVDF, Merck Millipore Inc., Darmstadt, Germany) membrane using the electrophoresis unit (Bio-Rad Laboratories, Inc., Hercules, CA, USA). The membranes were blocked with 5% skim milk in TBS-T at room temperature for 1 h and washed 10 min three times with TBS-T. The PVDF membrane was incubated with primary antibodies overnight at 4 °C and then with HRP-conjugated goat anti-rabbit (cat. no. SA00001-2, 1:10,000 dilution) or goat anti-mouse IgG (cat. no. SA00001-1; 1:10,000 dilution) (ProteinTech Group, Inc.) for 1 h at room temperature. All proteins were detected using an ECL Western blotting kit (SB-WB012, Shanghai Shenger Biotechnology Co., LTD). The following antibodies were used in this study: KIF22 (Cat No: 13403-1-AP, 1:2000 dilution), CDC25C (Cat No: 16485-1-AP, 1:5000 dilution), CDK1 (Cat No: 19532-1-AP, 1:1000 dilution), cyclinB1 (Cat No: 55004–1-AP, 1:2000 dilution), GAPDH (Cat No: 10494-1-AP, 1:10000 dilution) (ProteinTech Group, Inc.), p-ERK (301245, 1:1000 dilution, ZEN BIO, China), T-ERK(250338, 1:1000 dilution, ZEN BIO, China). Gray analysis was performed by the ImageJ software (National Institutes of Health, Bethesda, MD, USA). The level of protein expression was normalized according to GAPDH expression. Image J was used to calculate the grayscale (InDen) of all the protein bands and then subtract the IntDen of the image background. The gray value of the target protein is divided by the gray value of the internal reference band of the corresponding band, and the relative expression amount of the target protein in this group was taken (target protein /GAPDH).

### Cell viability assay

A Cell Counting Kit-8 (CCK-8) assay was used to detect the viability and proliferation of MM cells. After transfection of siRNA, RPMI 8226 (4,000 cells/well) and MM.1S cells (7,000 cells/well) were seeded in three duplicates in 96-well plates. RPMI 8226 (5,000 cells/well) and OPM2 (5,000 cells/well) were seeded in three duplicates in the 96-well plates after overexpressing KIF22. A CCK-8 proliferation assay kit (Shanghai Qihai Futai Biotechnology Co., Ltd.) was used according to the manufacturer's instructions.

### 5-Ethynyl-20-deoxyuridine (EdU) incorporation assay

The proliferation of MM cells was investigated using a BeyoClick™ EdU Cell Proliferation Kit with Alexa Fluor 555 (C0075S, Beytime, China). Briefly, MM cells transfected with siRNA were seeded in the 24-well plates for 72 h, and 1 μL of 10 μM EdU medium was added to each well. The cells were then incubated for 2 h and fixed with 4% paraformaldehyde. Then, after being permeabilized with 0.5% Triton X-100, cells were stained with a click reaction solution containing Azide and CuSO_4_ at room temperature L for 30 min. EdU-labeled cells were counted by flow cytometry, and cell proliferative activity was detected by the percentages of EdU-positive cells.

### Cell cycle assay

Following the instructions for the Cell Cycle and Apoptosis Analysis Kit (C1052, Beytime, China), RPMI 8226, MM.1S, and OPM2 cells were collected 48 h after transfection, washed twice with cold PBS, then suspended in cold 70% ethanol and fixed overnight at 4 °C. Then, the cells were washed twice in cold PBS and suspended in 300 μL PBS containing 40 μg/mL RNase A and incubated for 30 min at room temperature before 300 μL staining solution (50 μg/mL PI in PBS/5 mM EDTA) was added. Cell cycle analysis was detected by a BD FACSCanto II flow cytometer (BD Biosciences) within 24 h. The data were analyzed by FlowJo version 10 (BD Biosciences).

### Cell apoptosis

Annexin V–PE/RedNucleus II assay by Shanghai Bioscience, China, and Annexin V–APC/7-AAD Apoptosis Kit by Pricella, Wuhan, China were used. The MM cell lines were transfected with siRNA and cultured in 24-well plates (1 × 10^5^/ well). After 72 h, 5 μL Annexin V–PE and 5 μL RedNucleus II were added and cultured in the dark at room temperature for 20 min. The apoptosis of MM cells was detected by flow cytometry.  Another Annexin V–APC/7-AAD Apoptosis Kit was used for MM cell lines transfected with KIF22 ovexpression lentivirus. The same procedure as above was used. The results were analyzed by FlowJo software.

### Cell migration and invasion assay

To determine the migration and invasion of MM cells, we used a 24-well culture plate and Transwell chamber (Corning Corporation). In the migration experiment, MM cells (5 × 10^5^) were washed once in serum-free medium and then inoculated on a rehydrated underlying membrane with a diameter of 8 μm. The lower cavity was filled with a complete medium containing 20% FBS. For the invasion experiment, Matrigel (BD Biosciences) was pre-coated overnight in the upper chamber of the Transwell chamber at 37 °C, other steps being the same as in the migration experiment. After incubation at 37℃ and 5% CO_2_ for 24 h, cells in the lower chamber were collected and counted by flow cytometry. The cells were counted three times and all tests were repeated at least three times.

### Chromatin immunoprecipitation (ChIP) assay

ChIP experiments were conducted with the ChIP Assay Kit (P2078, Beyotime, Shanghai, China) according to the manufacturer's protocol. 1 × 10^6^ RPMI 8226 cells was fixed with 1% formaldehyde, re-suspended by a lysis buffer, and cleaved by ultrasound to shear the DNA into a range of 500–1,000 bp. The DNA and protein complex were then immunoprecipitated with anti-KIF22 antibody (Cat No:13403-1-AP) for 4 h at a room temperature, and the complex was enriched using protein A agarose (Beyotime Institute of Biotechnology). Magnetic beads were isolated and washed. Isolated DNA was purified using a DNA Purification Kit (D0033, Beyotime, Shanghai, China) Precipitated genomic DNA was amplified by RT-qPCR with the CDC25C promoter primers as follows:

forward, 5′- GGCACGAGAAAGAAGCGAAGA-3′,

reverse, 5′- CCGCCAGCCCAGTAACCTA -3′.

### Luciferase reporter gene assay

Luciferase reporter gene assay was conducted to directly analyze the transcriptional regulation of CDC25C by KIF22. The promoter of CDC25C containing wild type and mutant type were cloned in pGL3 vectors separately by transfection reagent (Lipoferter, Hanheng, China) and the sequences introduced in supplementary materials. 293T cells were cultured for 24 h and luciferase was measured with luciferase reporter gene assay kit (11401ES76, YEASEN Biotechnology Co., LTD, China). Results were normalized by firefly luciferase activity.

### Subcutaneous xenograft model in nude mice

Male nude mice (4 weeks old) were purchased from Chengdu Yaokang Biotechnology Co., LTD with the approval of the Ethics Committee of the Second Affiliated Hospital of Xi'an Jiaotong University (No. 20221601) and housed at the pathogen-free condition. Each group (*n* = 3) was injected 1 × 10^7^ Lv-NC RPMI 8226 cells and Lv-shKIF22 RPMI 8226 cells. MM cells were previously mixed with matrigel at a ratio of 1:3 (40183ES08, Yeasen Biotechnology Co., Ltd, China). We then used the same method to inject NC RPMI 8226 cells and OE-KIF22 RPMI 8226 cells into another group of mice. When the diameter of subcutaneous tumor was more than 15 mm, mice were killed, and subcutaneous tumors were dissected for size measuring, weighing, and IHC assays.

### Immunohistochemistry (IHC) staining

Paraffin-embedded sections of subcutaneous tumor tissue were obtained. Paraffin-embedded slides and tissues were dewaxed with xylene (I and II) and gradient alcohols (100%, 95%, 85%, and 75%). Antigen retrieval was performed by microwave sectioning in citrate buffer for 2 min. The slides were incubated with 3% hydrogen peroxide (H_2_O_2_) at room temperature for 20 min and goat serum at room temperature for 30 min. The primary antibody was incubated at a suitable concentration of 4 ℃ overnight. Biotinylated anti-IgG was added and incubated at room temperature for 1 h. Streptomycin–HRP was incubated for 30 min, stained with DAB, and then stained with hematoxylin. After rinsing with running water, the slides were dehydrated with gradient alcohol and xylene and finally sealed with neutral gum. Primary antibodies included KIF22 (13403-1-AP, 1:200, proteintech), CDC25C (16485-1-AP, 1:500, proteintech), Ki-67(28074-1-AP, 1:500, proteintech), and CD38(25284-1-AP, 1:300, proteintech).

### Statistical analysis

We used *t* test to detect the correlation between KIF22 expression and clinical characteristic data. Kaplan–Meier curve was used for survival analysis and the univariate survival analysis. The *t* test and Mann–Whitney *U* test were used to compare the different groups. For the comparison of multiple groups, Kruskal–Wallis test was used for non-parametric data, while ANOVA followed by Tukey post hoc test was used for parametric data. Data are recorded in the form of mean ± SD. Statistical analyses were performed with Prism 8 (GraphPad Software) and SPSS 24 (SPSS Inc.). *P* < 0.05 was considered to be statistically significant difference.

## Results

### KIF22 is highly expressed in MM patients and associated with poor prognosis

**Fig. 1:**
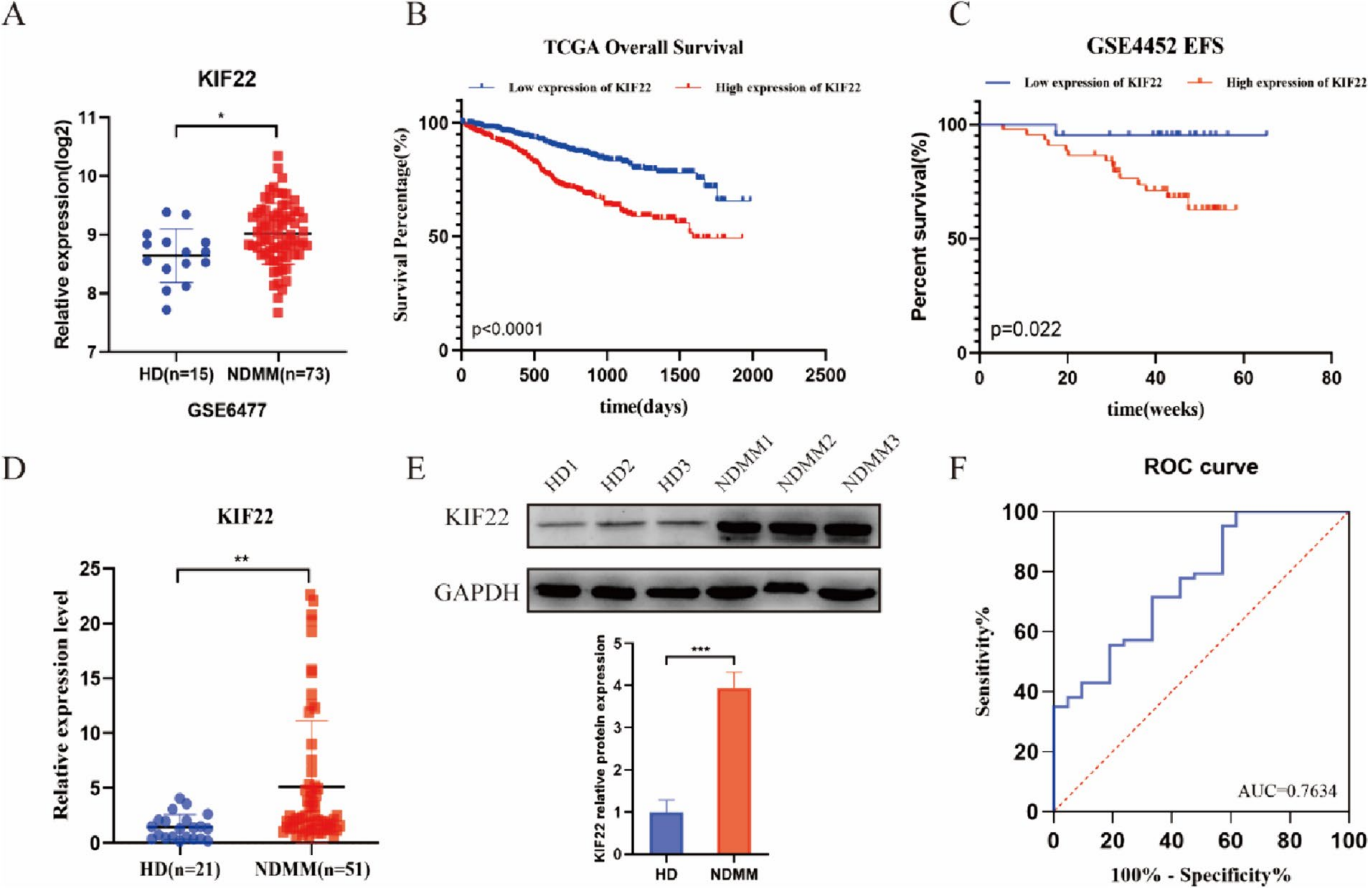
KIF22 was highly expressed in MM and associated with the prognosis of MM patients. **A** The expression of KIF22 was analyzed in patients with newly diagnosed MM and healthy donors from the GEO database (GSE6477). **B** Overall survival data of MM patients were collected from TCGA GDC MMRF-COMMPASS database and then analyzed by the Kaplan–Meier plot. **C** Event-free survival data of MM patients were collected from GSE4452 and analyzed by the Kaplan–Meier plot. **D** The mRNA expression of KIF22 was detected by the specimens from MM patients and healthy donors. **E** The protein KIF22 expression of NDMM patients were detected by Western blotting. **F** ROC curve based on KIF22 expression in NDMM patients and healthy donors. vs. respective control. *NDMM* newly diagnosed multiple myeloma; *P < 0.05, **P < 0.01, ***P < 0.001

The expression of KIF22 was analyzed between newly diagnosed MM patients and healthy donors in the GSE6477 dataset and we found that KIF22 was highly expressed in MM patients (Fig. 1A). To explore the relationship between the KIF22 expression and prognosis of MM, survival data of 858 MM patients were collected from the TCGA GDC MMRF-COMMPASS database and 65 MM patients from the GSE4452 dataset. Then we analyzed overall survival time and event-free survival time by Kaplan–Meier plotter (Fig. 1B, C). The results revealed that higher expression of KIF22 was significantly associated with a shorter OS (*P* < 0.0001) and EFS (*P* = 0.022) for MM patients, which indicated a poor prognosis.

A total of 72 specimens consisting of 51 newly diagnosed MM (NDMM) patients and 21 healthy donors were collected, the basic features of which are displayed in Table 1. We found that the mRNA as well as protein expression levels of KIF22 was upregulated in MM patients compared with healthy donors, which was consistent with the analysis of the public datasets (Fig. 1D, E). Then we plotted ROC curves based on KIF22 expression in patients and healthy donors. ROC curve analysis demonstrated that the area under the curve (AUC) values was 0.7634, indicating the potential value of KIF22 in distinguishing MM patients from healthy donors.

Clinically, the correlation between the expression of KIF22 and clinical characteristics was analyzed. Results indicated that the gender (*P* = 0.016), level of LDH (*P* < 0.001), β_2_-MG (*P* = 0.003), and percentage of tumor cells (BM) (*P* = 0.002) in MM patients were correlated with the expression of KIF22 (Table 1). It revealed that MM patients with high tumor burden had higher expression of KIF22. However, there was no significant associations which were observed between KIF22 expression and other clinicopathological parameters, such as age, hemoglobin, albumin, D-S stage, and ISS stage and R-ISS stage.

### KIF22 regulates proliferation and cell cycle of MM cells in vitro

**Fig. 2:**
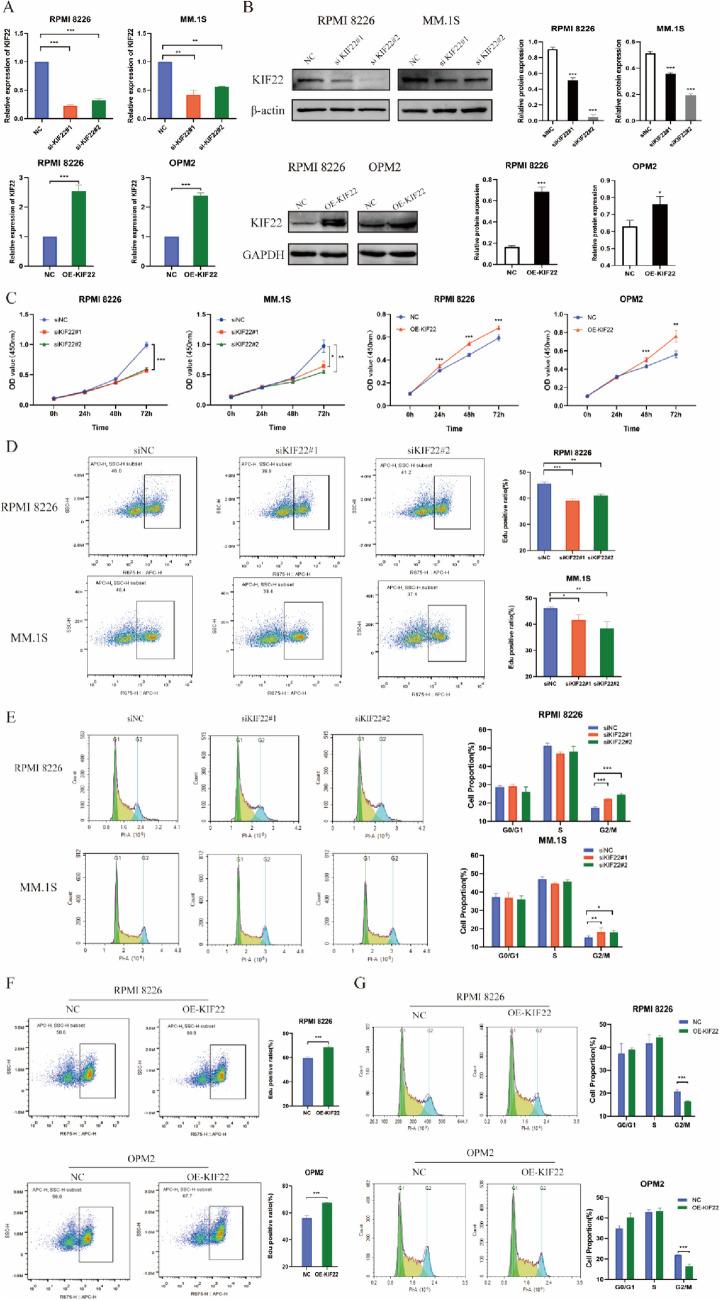
KIF22 regulated proliferation and cell cycle of MM cells in vitro. **A–B** Efficiency of knockdown of KIF22 in mRNA and protein levels in MM cells. **C** Cell proliferation was detected in KIF22-knockdown and KIF22-overexpression MM cells by the CCK-8 assay. **D–E** 5-Ethynyl-20-deoxyuridine (EdU) incorporation assay and cell cycle results in MM cells after knockdown of KIF22. **F–G** EdU incorporation assay and cell cycle results in MM cells after the overexpression of KIF22. *P < 0.05, **P < 0.01, ***P < 0.001

The expression of KIF22 was detected in different MM cell lines, and feasible cell lines were selected for experiments in vitro (Fig. S1). We used siRNA to knock down the expression of KIF22 in MM cell lines, including RPMI 8226 and MM.1S to explore the biological function of KIF22 in MM. KIF22-overexpression cell lines were conducted by transfection of lentiviral vector in RPMI 8226 and OPM2. RT-qPCR and Western blotting results showed that KIF22 was significantly silenced in MM.1S and RPMI 8226 cells, and overexpressed in RPMI 8226 and OPM2 cells (Fig. 2A, B). Then CCK-8 assay results showed that, compared with the siNC group, MM cell viability was inhibited (Fig. 2C) in siKIF22 group cells and was significantly enhanced in the OE-KIF22 group. Besides, EdU assays also indicated that proliferation of MM was suppressed after silencing KIF22 (Fig. 2D). Further, the role of KIF22 in MM cell cycle regulation was investigated. As expected, we found that KIF22 silencing significantly increased the proportion of MM cells in the G2/M phase and caused cell cycle arrest at the G2/M phase (Fig. 2E).

Meanwhile, EdU assays indicated that proliferation of MM was promoted in KIF22-overexpression MM cells compared to NC group cells (Fig. 2F), and KIF22-overexpression MM cells had a decreased proportion of G2/M phase compared with NC group cells (Fig. 2G). Taken together, the high expression of KIF22 could promote proliferation of MM cells and G2/M cell cycle transition.

Besides, we also detected the effect on apoptosis, migration, and invasion of MM cells by flow cytometry after changing the expression of KIF22. However, in MM cell lines, there was no significant difference of apoptosis between KIF22-knockdown or KIF22-overexpression group and control group. Migration and invasion of MM cells were performed by Transwell assays and flow cytometry. We also observed that the expression of KIF22 had no significant effect on the migration and invasion of MM cells (Fig. S2-S3).

### KIF22 positively transcriptionally regulated the expression of CDC25C by directly binding its promoter

**Fig. 3:**
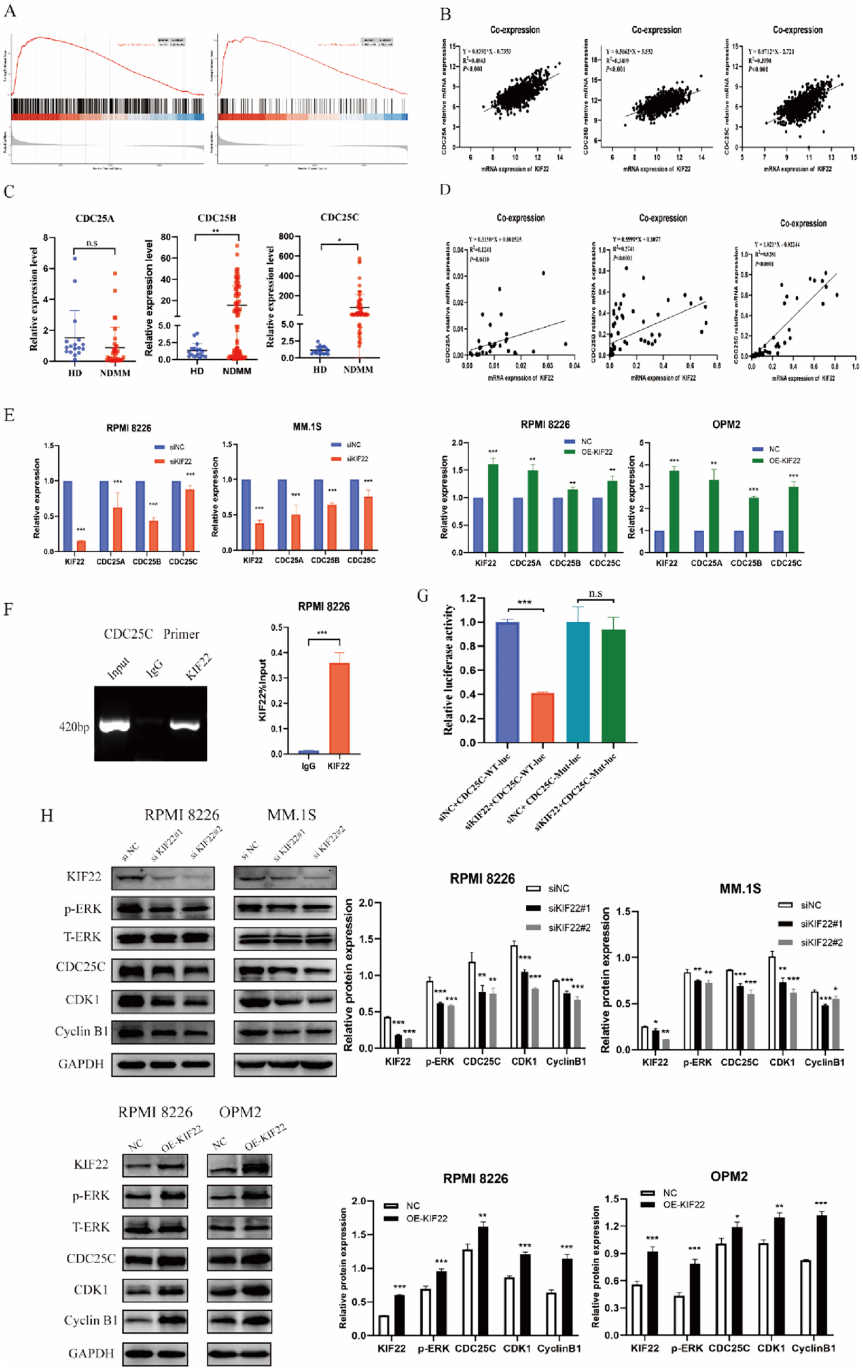
KIF22 transcriptionally regulated the expression of CDC25C. **A** Results of an enrichment for regulation of mitotic cell cycle and cell cycle G2/M phase transition, as shown by performing GSEA. **B** Co-expression analysis performed by TCGA data between KIF22 and CDC25A, CDC25B, and CDC25C. **C** The mRNA expression of KIF22 was detected by the specimens from MM patients and healthy donors. **D** Co-expression analysis performed by data of  MM patients samples between KIF22 and CDC25A, and CDC25B and CDC25C. **E** The mRNA expressions of CDC25A, CDC25B, and CDC25C were detected in MM cells after the knockdown or overexpression of KIF22. **F** PCR amplification of the anti‑KIF22 antibody‑enriched CDC24C promoter fragment in RPMI 8226 cells by ChIP assay. **G** Luciferase activity of pGL3‑CDC25C-Mut, pGL3‑CDC25C-WT  plasmids in 293 T cells co‑transfected with siKIF22 or siNC examined by luciferase reporter assays. **H** The ERK/CDC25C/CDK1/cyclin B1 pathway was detected in MM cells after the knockdown or overexpression of KIF22. *Mut* mutation type. *WT* wild type. *P < 0.05, **P < 0.01, ***P < 0.001. *n.s* no significance

To explore the possible phenotypic function of KIF22, we firstly performed GSEA analysis, which indicated that the function of KIF22 was mainly involved in the regulation of mitotic cell cycle and cell cycle G2/M phase transition (Fig. 3A). At the same time, both KEGG analysis and GO analysis showed that the expression of KIF22 mainly affected the cell cycle and its regulation (Fig. S4). Therefore, we hypothesized that the expression of KIF22 mainly affects MM cell proliferation and cell cycle.

To clarify the underlying mechanism of KIF22-affected cell cycle, we focused on the G2/M checkpoint-related protein markers. Intriguingly, we found that the mRNA expression of CDC25A, CDC25B, and CDC25C were all positively correlated with the expression of KIF22 by analyzing the TCGA data (Fig. 3B). Then RT-qPCR results showed that CDC25B and CDC25C were highly expressed in NDMM patients compared with healthy donors (Fig. 3C). The results of co-expression analysis in the MM patient samples revealed that CDC25A, CDC25B, and CDC25C were all positively correlated with KIF22 expression, especially CDC25C had the strongest linear fitting correlation with KIF22 (Fig. 3D). Subsequently, RT-qPCR results showed that the expression of CDC25A, CDC25B, and CDC25C was downregulated in siKIF22 MM cells and upregulated in KIF22-overexpression MM cells, which was consistent with the analysis of the database. (Fig. 3E).

Additionally, it has been studied that CDC25A mainly activated the CDK2/cyclin E and CDK2/cyclin A complexes during the G1/S transition. CDC25B is proposed to be responsible for the initial activation of CDK1/cyclin B1 at the centrosome during the G2/M transition, which is then followed by a complete activation of CDK1/cyclin B1 complexes by CDC25C in the nucleus at the onset of mitosis (Boutros et al. 2007). Mechanistically, some studies had revealed that KIF22 could act as a transcription factor to regulate the expression of downstream genes, and KIF22 might bind to the centromere DNA sequence through an interaction with the alphoid sequence of 5′-CCACTCCCAGACTT-3′ (Tokai et al. 1996). Considering that KIF22 regulated cell cycle G2/M phase in our study, we respectively designed primers of promoter of CDC25B and CDC25C and performed the ChIP assay. The results showed that KIF22 could only bind to the promoter of CDC25C and positively regulate its transcription, which was identified by nucleic acid electrophoresis and RT-qPCR instead of CDC25B (Figs. 3F and S5). Then we performed luciferase reporter gene assay to verify the interaction between KIF22 and CDC25C. The promoter of CDC25C containing wild type and mutant type were cloned in pGL3 vectors and co-transfected with siNC or siKIF22 into 293T cells. The result revealed that the luciferase activity of wild type with siNC was significantly higher than that with siKIF22 and there was no significance in luciferase activity between the siNC and siKIF22 groups in the mutant type of CDC25C (Fig. 3G). Thus, the result confirmed that KIF22 could transcriptionally regulate the expression of CDC25C.

On the other hand, Western blotting results showed that the ERK/CDC25C/CDk1/cyclinB1 pathway was inhibited in KIF22-knockdown MM cells, but activated in KIF22-overexpression MM cells (Fig. 3H), which indicated that KIF22 could regulate the ERK/CDC25C/CDk1/cyclinB1 pathway by transcriptional regulation of CDC25C.

### The inhibitor of CDC25C suppresses cell proliferation and causes cell cycle arrest in MM cells

**Fig. 4:**
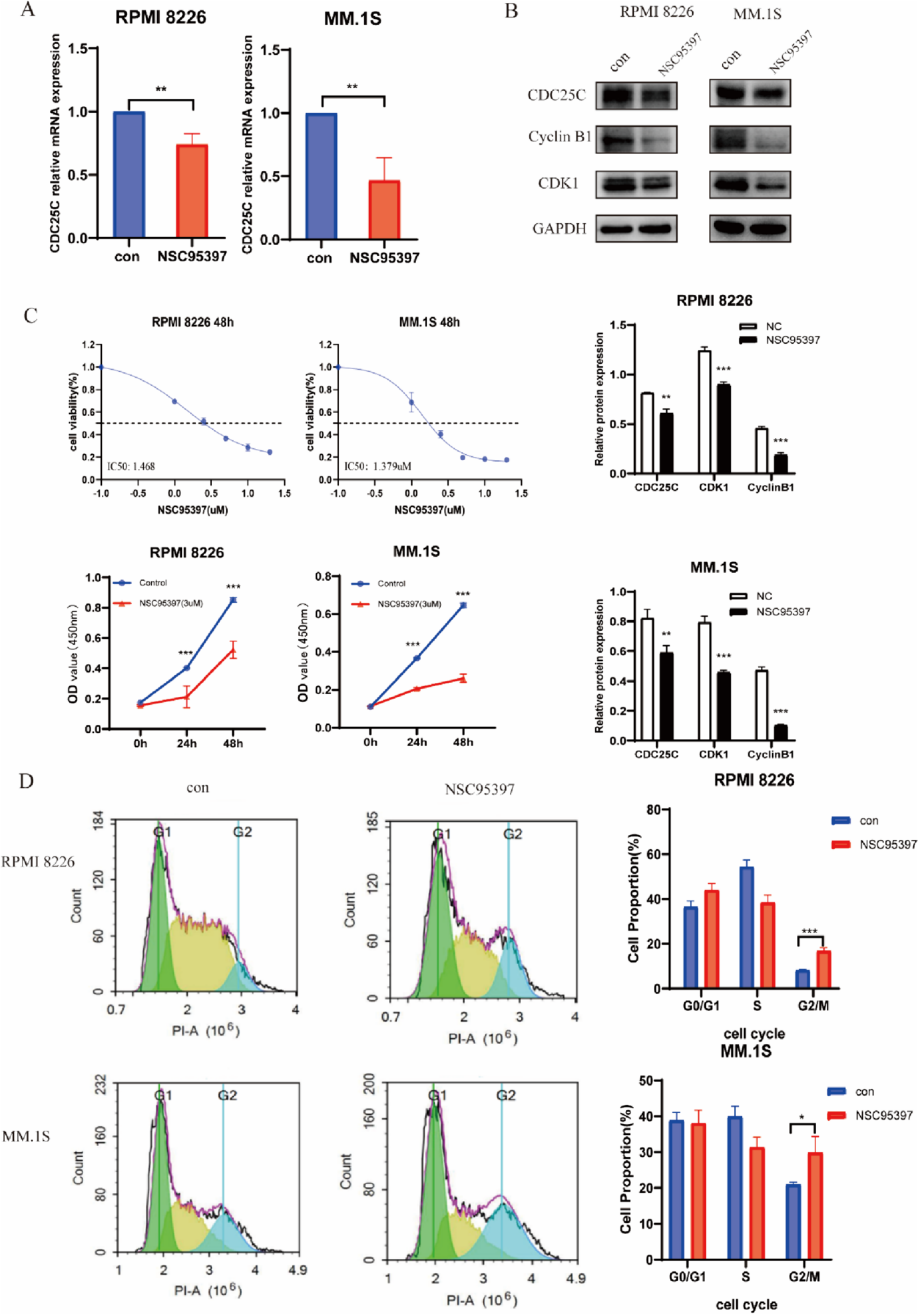
CDC25C inhibitor suppressed proliferation and arrested cell cycle of MM cells. **A** Efficiency of CDC25C inhibitor NSC95397 on suppressing the expression of CDC25C. **B** The CDC25C/CDK1/cyclinB1 pathway was inhibited after using CDC25C inhibitor in MM cells. **C**  CCK-8 assay was used to determine the appropriate concentration of NSC95397 on MM cells and its effect on MM cell proliferation.  **D** CDC25C inhibitor arrested MM cells at the G2/M phase. *P < 0.05, **P < 0.01, ***P < 0.001

Furthermore, to investigate the function of CDC25C in MM cells, its inhibitor NCS95397 was used. First, we determined the efficiency of NSC95397 for inhibiting the expression of CDC25C in mRNA and protein levels (Fig. 4A, B). Then pre-experiment was conducted CCK-8 assay to determine the appropriate concentration of NSC95397 for MM cells (Fig. 4C). Next, CCK-8 assay was performed and we found that the proliferation was inhibited and the cell cycle was arrested at the G2/M phase as well when MM cells were treated with NSC95397 (Fig. 4D). Additionally, the CDC25C/CDk1/cyclinB1 pathway was also suppressed by NSC95397 in MM cells (Fig. 4B).

### The CDC25C inhibitor NSC95397 partially inhibited MM cell proliferation and arrested cell cycle caused by the overexpression of KIF22

**Fig. 5:**
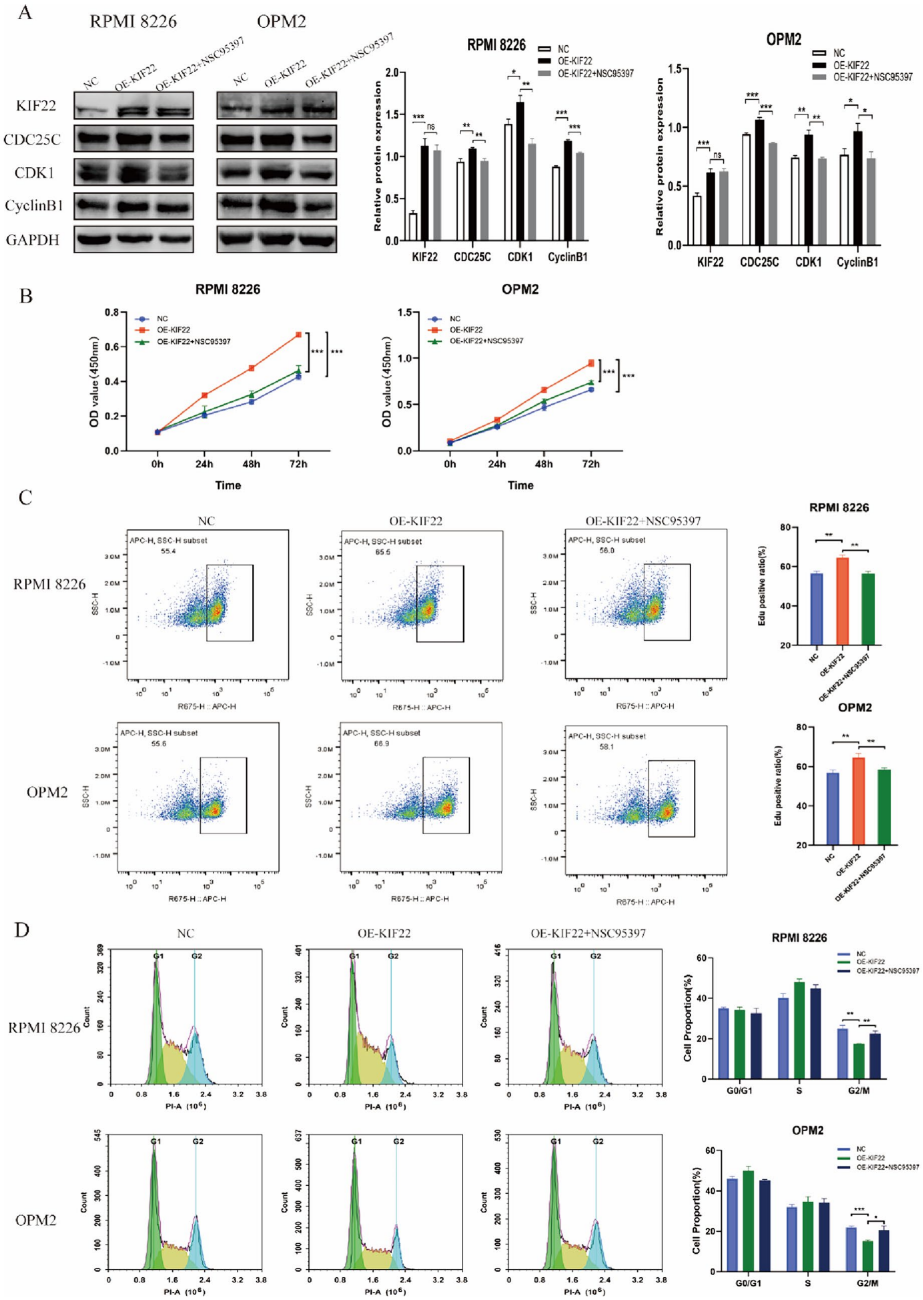
KIF22 regulates MM progression through the CDC25C/CDK1/cyclinB1 pathway. **A** The CDC25C/CDk1/cyclinB1 pathway was detected by Western blotting in the overexpression of KIF22 MM cells treated with NSC95397. **B–C** The CCK-8 and 5-ethynyl-20-deoxyuridine (EdU) incorporation assay results in the overexpression of KIF22 MM cells treated with NSC95397. **D** Cell cycle results in the overexpression of KIF22 MM cells treated with NSC95397. *P < 0.05, **P < 0.01, ***P < 0.001

Our results showed that the highly expressed KIF22 in MM cells activated the CDC25C/CDk1/cyclinB1 pathway by transcriptionally regulating CDC25C to promote proliferation and cell cycle transition. To assess whether the CDC25C inhibitor NSC95397 could reverse KIF22-mediated regulation of proliferation and cell cycle transition by inhibiting the CDC25C/CDk1/cyclinB1 pathway, we treated KIF22-overexpressed MM cells with NCS95397. Western blotting results showed that the upregulation of CDC25C/CDk1/cyclinB1 pathway caused by KIF22 overexpression in MM cells was inhibited by NSC95397 (Fig. 5A). The results of CCK-8 assay and EdU assay both demonstrated that NSC95397 significantly inhibited the proliferation caused by KIF22 overexpressing in MM cells (Fig. 5B, C). Furthermore, the proportion of G2/M phase cells was increased when KIF22-overexpression MM cells were treated with NSC95397. These results further demonstrated that KIF22 regulated MM progression by regulation of CDC25C.

### The expression of KIF22 influences the growth of subcutaneous tumor in nude mice

**Fig. 6:**
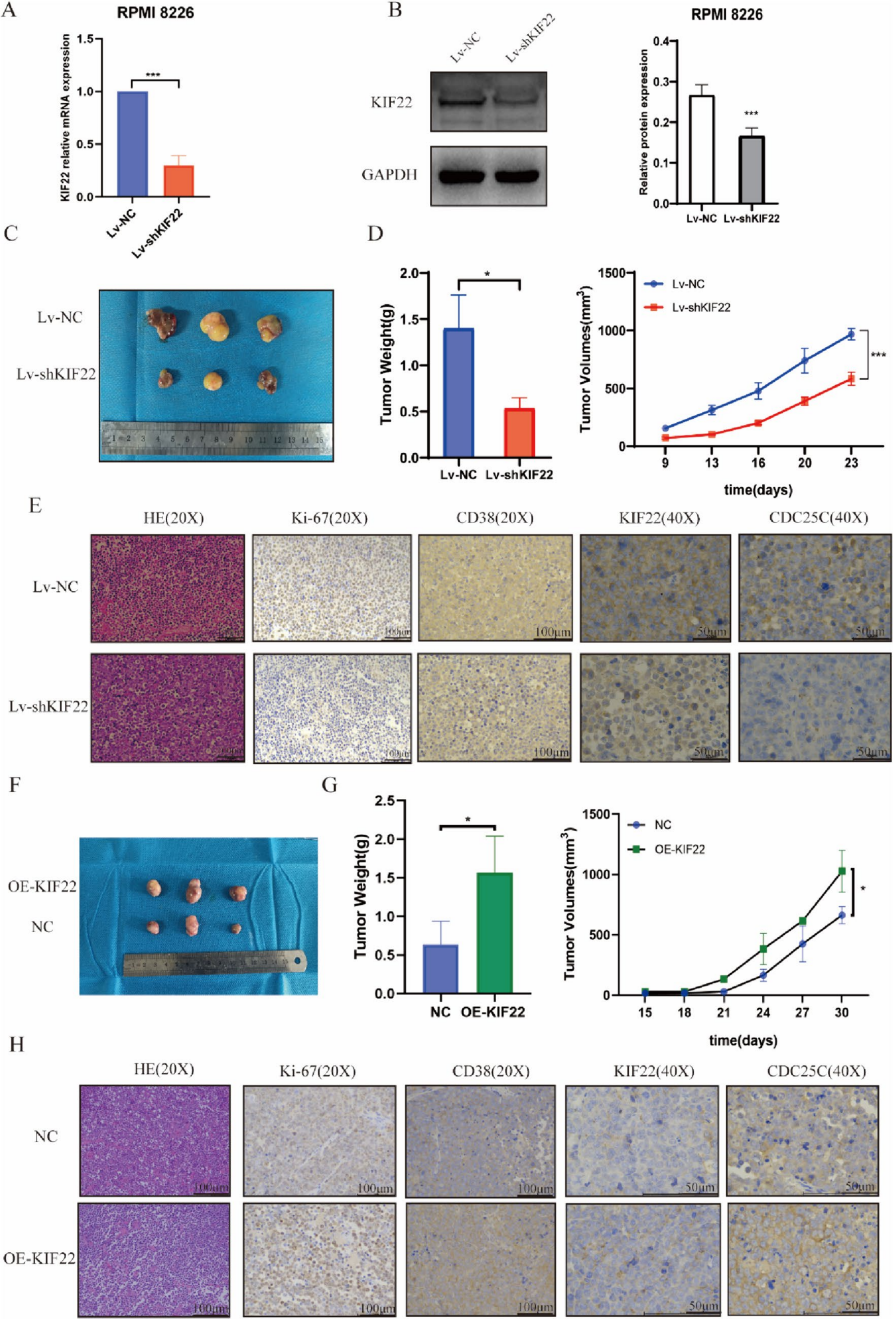
Knockdown of KIF22 suppressed the growth of subcutaneous tumor in nude mice. **A-B** Efficiency of KIF22 knockdown was detected in mRNA and protein level. **C** Representative images of tumors formed in the Lv-NC group and Lv-shKIF22 group. **D** Tumor volume and growth curves. **E** HE stain and immunohistochemistry revealed the expression of KIF22, CDC25C,Ki-67, and CD38 in the tumor tissues from mice. **F** Representative images of tumors formed in the NC group and OE-KIF22 group. **G** Tumor volume and growth curves. **H** HE stain and immunohistochemistry revealed the expression of KIF22, CDC25C, Ki-67, and CD38 in the tumor tissues of mice. *P < 0.05, **P < 0.01, ***P < 0.001

To investigate the function of KIF22 in MM growth in vivo, RPMI 8226 cells were transfected with shControl and shKIF22 lentivirus and the efficiency of knockdown was verified on both mRNA and protein levels (Fig. 6A, B). Subcutaneous xenograft nude mice models were constructed with Lv-NC RPMI 8226 cells and Lv-shKIF22 RPMI 8226 cells. The results indicated that the growth of tumor in the Lv-shKIF22 group mice was significantly inhibited compared with the Lv-NC group. In addition, the Lv-shKIF22 group mice had lighter weight and less volume of tumor than the Lv-NC group (Fig. 6C, D). IHC results revealed that KIF22, CDC25C, CD38, and Ki-67 were weakly stained in the Lv-shKIF22 group (Fig. 6E). Subsequently, we used the same method to inject NC RPMI 8226 cells and OE-KIF22 RPMI 8226 cells into another group of mice. The results revealed that the growth of tumor in the OE-KIF22 group mice was significantly accelerated compared with the NC group (Fig. 6F). The OE-KIF22 group mice had heavier weight and bigger volume of tumor (Fig. 6G), and IHC results suggested that KIF22, CDC25C, CD38, and Ki-67 were strongly stained in the OE-KIF22 group (Fig. 6H). To sum up, the expression of KIF22 could influence MM cell proliferation and tumor burden in vivo.

## Discussion

It is generally accepted that cytogenetic abnormalities and bone marrow microenvironment are mainly involved in the oncogenesis and progression of MM (Holthof and Mutis 2020). Somatic mutations related to the development of MM can be divided into non-hyperdiploid disease (NHD) and hyperdiploid disease (HD) (Walker et al. 2010). It leads to changes in chromosome structure and dysregulation of cell cycle (Chng et al. 2006; Walker et al. 2010), which then results in the initiation and development of MM.

KIFs participate in several essential cellular functions, including mitosis, meiosis, and transport of macromolecules (Goldstein and Philp 1999; Hirokawa et al. 2009; Niwa 2015). A number of KIF proteins have been demonstrated to have an aberrant overexpression in various cancers and to be associated with malignant degree as well as drug resistance (Li et al. 2019; Lucanus and Yip 2018). It has been reported that KIF20B promoted the progression of clear cell renal cell carcinoma, and KIF18B promotes cell proliferation in colon adenocarcinoma (Li et al. 2019; Zhao et al. 2020). Studies showed that KIFC1 and KIF2A were both highly expressed in breast cancer and promoted the proliferation and migration of breast cancer cells (Li et al. 2020a; Li et al. 2015; Wang et al. 2014). Besides, KIF2C/4A/10/11/14/18B/20A/23 were also reported to be associated with the poor prognosis and promoted cell proliferation in hepatocellular carcinoma (Li et al. 2020b).

KIF22, as a member of KIFs, has been reported to be of high expression in a number of cancers. However, its role and function in MM still remain unclear. In our study, public datasets and clinical sample analysis demonstrated that the expression of KIF22 was higher in MM patients compared with healthy donors and correlated with poor prognosis. Then, knockdown of KIF22 in MM cells significantly inhibited the proliferation and caused G2/M phase cell cycle arrest, consistent with previous studies showing that KIF22 could influence the development of cancers such as pancreatic cancer (Zhang et al. 2022), breast cancer (Yu et al. 2014) and gastric cancer (Yu et al. 2020) by promoting cell proliferation and cell cycle dysfunction.

Previous studies have shown that the deficiency of KIF22 could induce abnormal mitosis and lead to inhibited proliferation by promoting cell cycle arrest. Our results indicated that KIF22 could bind to promotor of CDC25C to regulate its transcription in MM cells. CDC25C was also reported as a novel MAPK ERK 1/2 target (Cho et al. 2015), and ERK 1/2 bound and phosphorylated CDC25C. The suppression of ERK/CDC25C/CDK1/cyclin B1 pathway inhibited cell proliferation and induced G2/M phase arrest (Eymin et al. 2006). We found that the ERK/CDC25C/CDk1/cyclinB1 pathway was inhibited in KIF22-knockdown MM cells, but activated in KIF22-overexpression MM cells. Consequently, we speculated that KIF22 could indirectly regulate CDC25C through p-ERK. It has been reported that CDC25C was overexpressed in many cancers and correlated with prognosis. CDC25C was reported to be regulated by multiple mechanisms mainly including transcription regulation, in intracellular localization, interactions with partner proteins, and changes in phosphorylation levels and degradation (Boutros et al. 2007; St Clair et al. 2004). Researches have shown that CDC25C phosphorylated by CHK2 was unable to activate the CDk1/cyclinB1 complex, which promoted cell cycle arrest (Antoni et al. 2007; Chen et al. 2006; Wang 2014; Zannini et al. 2014). Aurora A promoted nuclear localization of CDC25C by phosphorylating polo-like kinase 1(PLK1) during mitosis (Bruinsma et al. 2014; Li et al. 2012; Toyoshima-Morimoto et al. 2002). Phosphoprotein phosphatase1 (PP1) dissociates and activates CDC25C from the cytoplasm to accelerate mitosis entry (Margolis et al. 2006, 2003; Perdiguero and Nebreda 2004). Subsequently, we found that CDC25C was highly expressed in MM patients and its inhibitor NSC95397 inhibited cell proliferation and caused G2/M phase cell cycle arrest, which confirmed that CDC25C played an important role in MM. Further experiments in MM cells overexpressing KIF22 indicated that KIF22 promoted MM progression by upregulation of CDC25C. Additionally, the promoting effect on proliferation of KIF22 overexpression could be reversed by NSC95397, indicating that the KIF22/CDC25C axis was  involved in regulating MM cell proliferation and cell cycle.

However, our study did not investigate the upstream regulation mechanism of KIF22. Previous studies have found that KIF22 could be regulated via both transcriptional and translational pathways. KIF22 was pre-transcriptionally regulated by miR-122 in esophageal squamous cell carcinoma cells (Wang et al. 2021). It was known that KIF22 could be phosphorylated by CDK1 to enhance its ability to bind to chromosomes during mitosis (Ohsugi et al. 2003). The mechanism of KIF22 regulating p-ERK has not been thoroughly studied, which we would further explore in the future.

Besides, in our clinical sample validation process, bone marrow mononuclear cells were extracted as MM cells, and plasma cell enrichment of bone marrow samples was not performed to verify our results because of the limited conditions. Subsequently, we will expand the samples and verify our existing experimental conclusions after plasma cells are sorted by flow cytometry.

## Conclusion

**Fig. 7:**
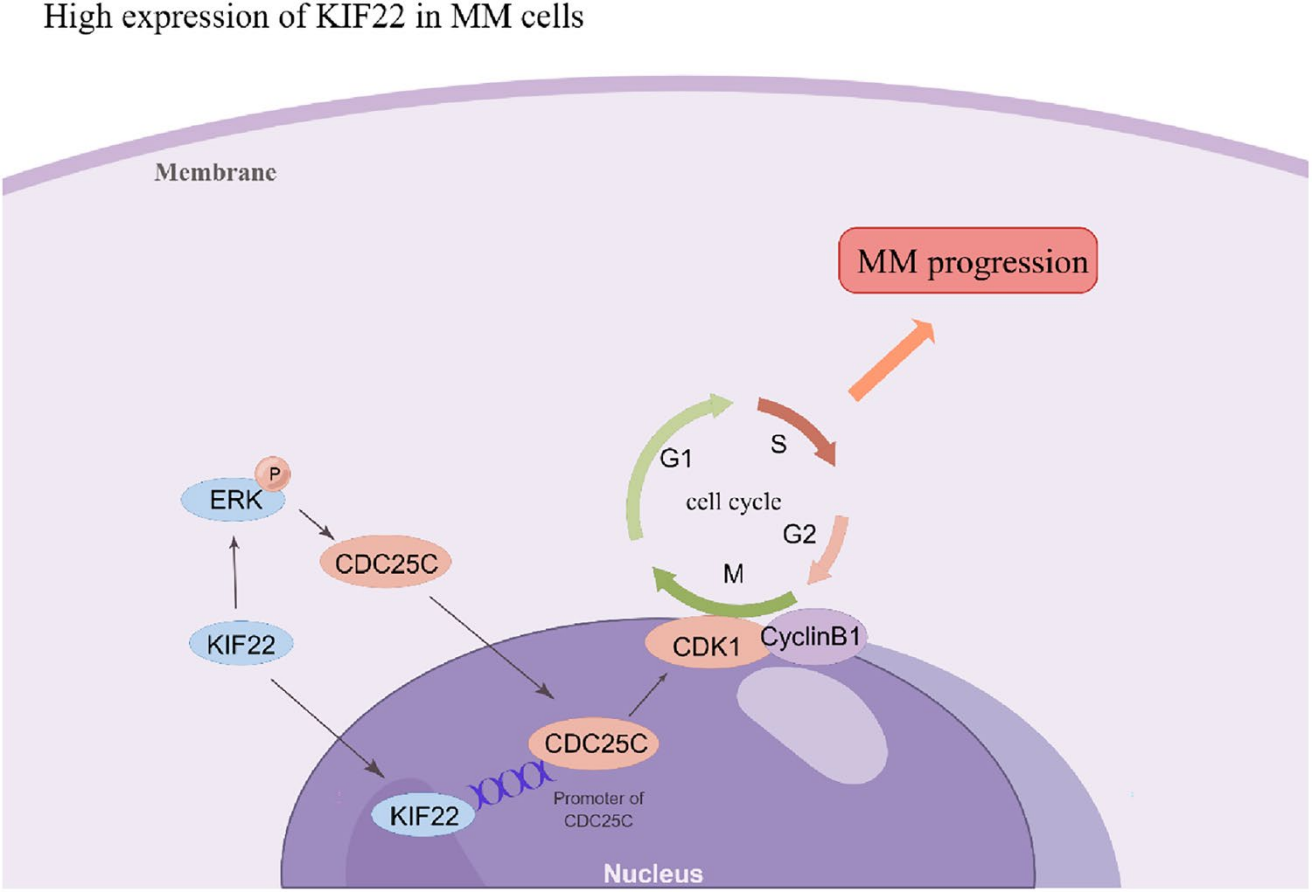
Mechanism diagram of KIF22 in regulating the progression of multiple myeloma (drawn by Figdraw). KIF22 positively transcriptionally regulated the expression of CDC25C by binding to its promoter and then regulated the CDC25C/CDk1/cyclinB1 pathway in MM cells

In conclusion, we demonstrated that KIF22 was highly expressed in MM patients and correlated with poor prognosis. Knockdown of KIF22 suppressed MM cell proliferation and caused G2/M phase cell cycle arrest, whereas overexpression of KIF22 showed the opposite effect. Mechanistically, we verified that KIF22 positively transcriptionally regulated CDC25C expression by binding its promoter, which in turn regulated the CDC25C/CDK1/cyclinB1 pathway in MM cells (Fig. 7). Therefore, our research provides a new insight into MM progression that KIF22 might be a promising marker and a potential therapeutic target in the future.

### Supplementary Information

Below is the link to the electronic supplementary material.

Supplementary file1 (DOCX 1733 kb)

## Data Availability

The original data supporting the conclusions of this study will be provided by the authors, and the data sets presented in this study can be found in online databases.
