# Supplementary material for: KIF22 promotes multiple myeloma progression by regulating the CDC25C/CDK1/cyclinB1 pathway
Source: J Cancer Res Clin Oncol. 2024 May 7;150(5):239. doi: 10.1007/s00432-024-05747-w (PMC11076398; doi:10.1007/s00432-024-05747-w)
Supplement: Supplementary file 1 — Supplementary file1 (DOCX 1733 kb) [file 432_2024_5747_MOESM1_ESM.docx]

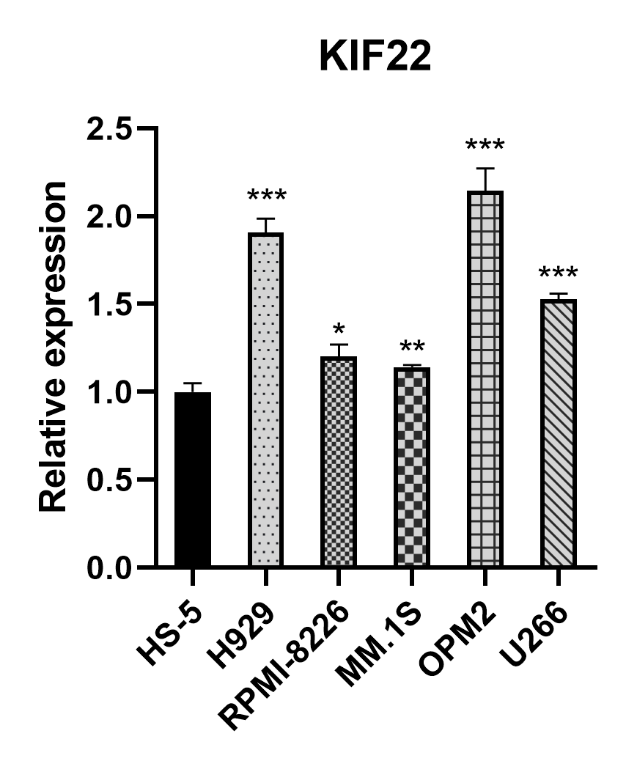


**Fig.S1** The expression of KIF22 was detected in different MM cell lines.


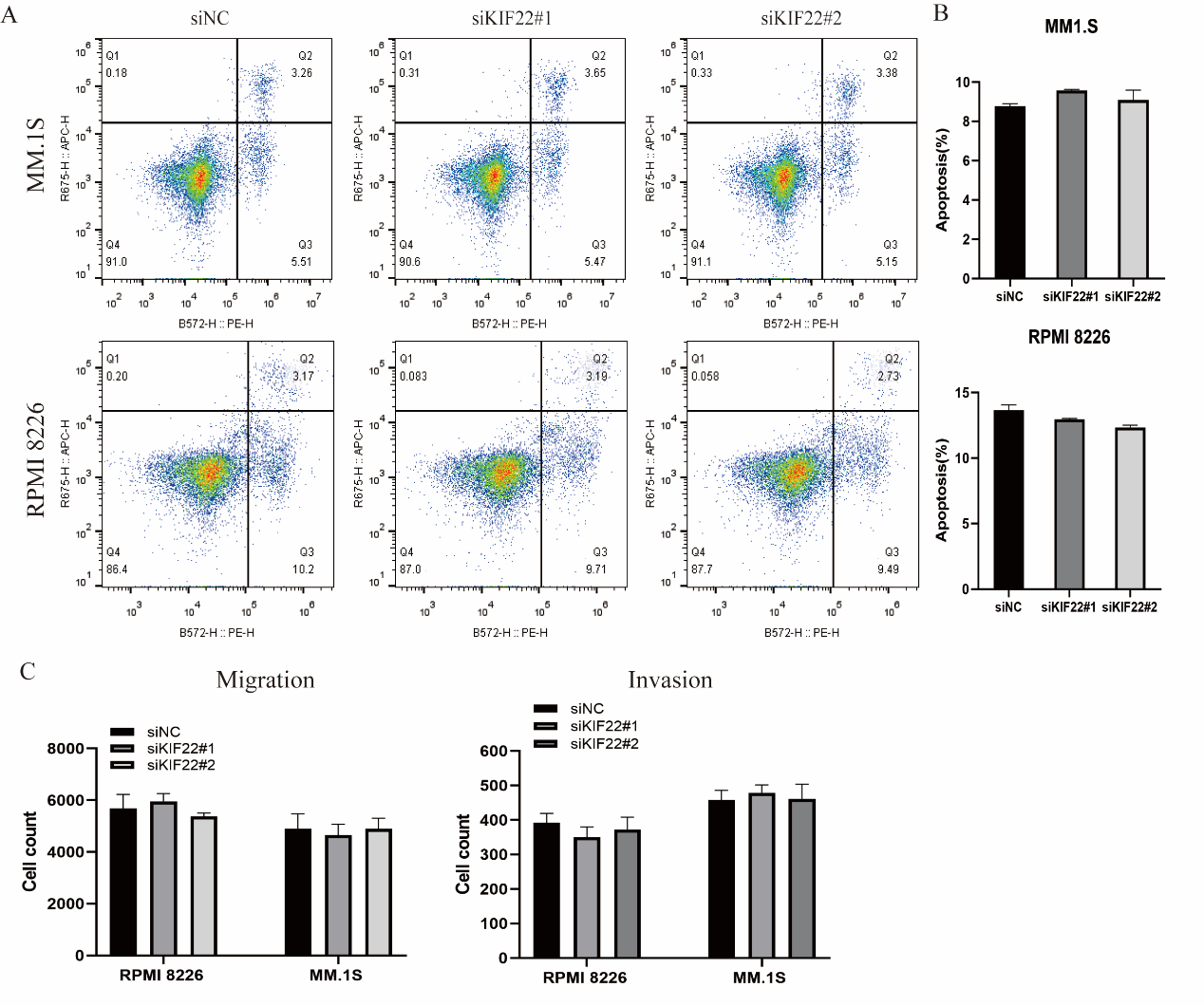


**Fig.S2** Effect of knocking down KIF22 in MM cells on apoptosis, migration and invasion. **A-B** Knockdown of KIF22 in RPMI 8226 and MM.1S cells had no effect on apoptosis. **C** Knocking down KIF22 in RPMI 8226 and MM.1S cells had no effect on migration and invasion.


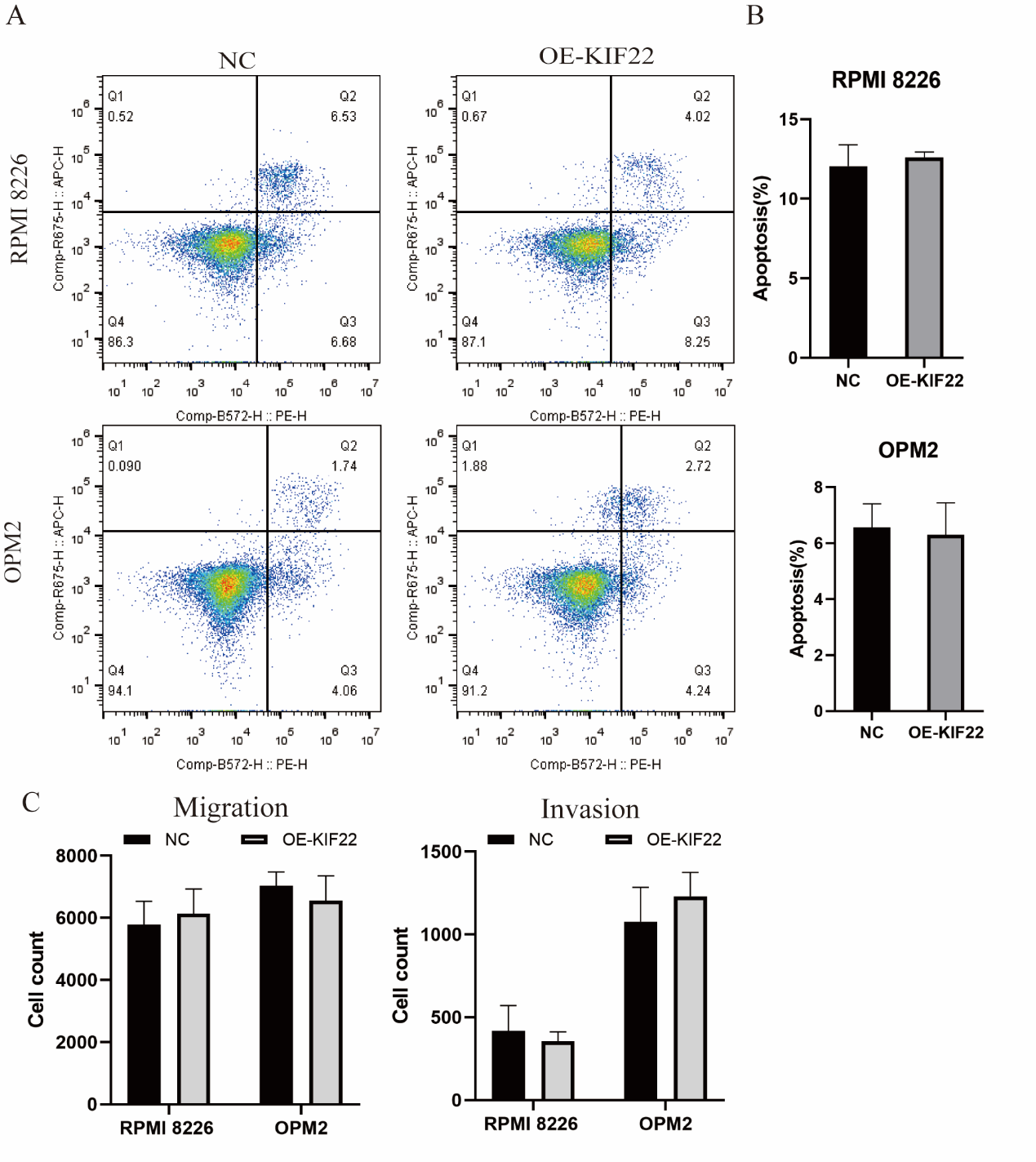


**Fig.S3** Effect of overexpression of KIF22 in MM cells on apoptosis, migration and invasion. **A-B** Knockdown of KIF22 in RPMI 8226 and MM.1S cells had no effect on apoptosis. **C** Knocking down KIF22 in RPMI 8226 and MM.1S cells had no effect on migration and invasion.


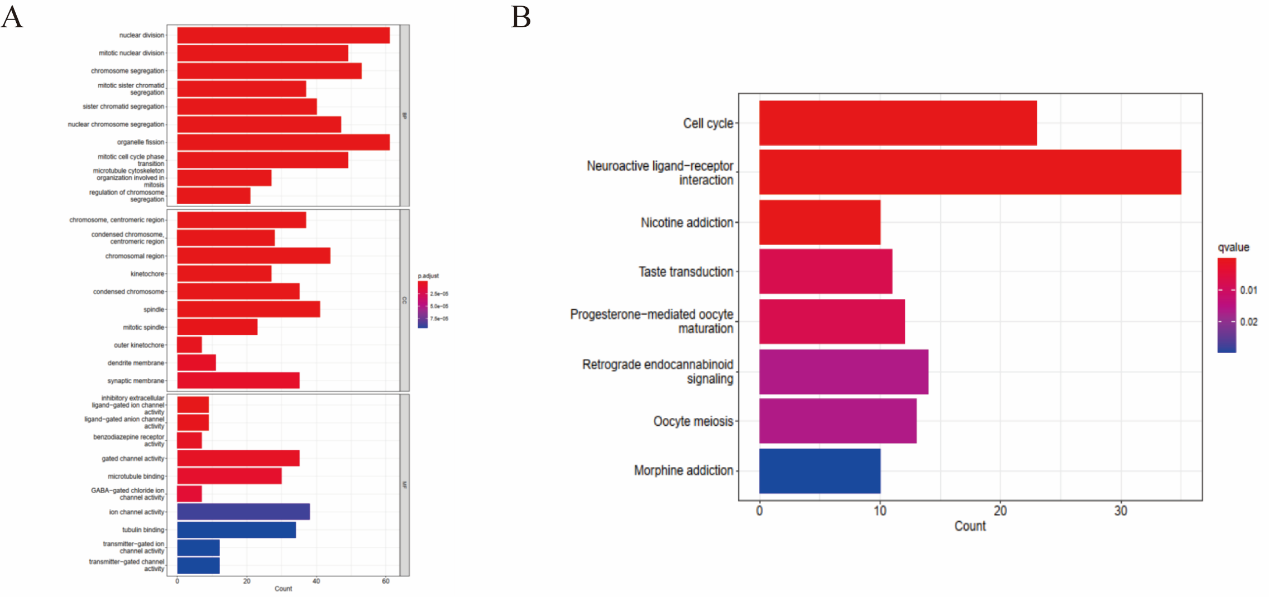


**Fig.S4** Differential gene enrichment analysis. **A** GO analysis. **B** KEGG analysis.


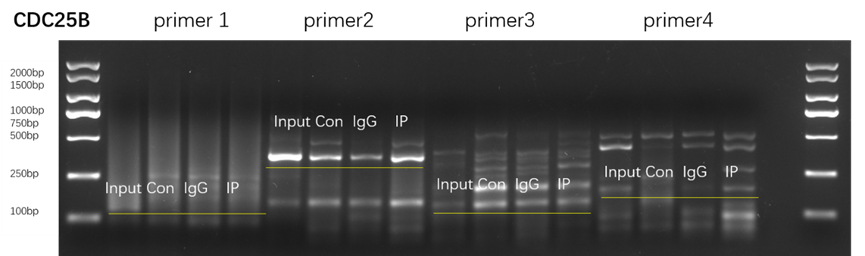


**Fig.S5**. ChIP result of CDC25B. We designed four primers according to the CDC25B promoter region, and no positive CHIP results were found.

Table.S1 The promoter sequence of wild type and mutant type of CDC25C

| Gene | Sequence |
| --- | --- |
| CDC25C promoter-WT | GGCACGAGAAAGAAGCGAAGAAAATGAAACGCTTACAACACCCTCCACCCAAAATAATCTCTCCTAGTATTACCCTAGTGAATGGACATCACTAGTAAGGCGCGGTTTAAATCTCCCGGGGTTCGTGGGGCTGAGGGAACGAGGAAAACAGAAAGGGTGTGGAGATTGGTGAGAGGGAGAGCCAATGATGCGCCAGGCTCCCCGTGAGGCGGAGCTTACCCCGCAGCCTGCCTAACGCTGGTGGGCCAAACACTATCCTGCTCTGGCTATGGGGCGGGGCAAGTCTTACCATTT**CCAGAGCA**AGCACACGCCCCCAGGTGATCTGCGAGCCCAACGATAGGCCATGAGGCCCTGGGCGCGCGCGCGGAGATTGGCTGACGCAGCTTAGAGGCGAGCGGGGATAGGTTACTGGGCTGGCGG |
| CDC25C promoter-Mut | GGCACGAGAAAGAAGCGAAGAAAATGAAACGCTTACAACACCCTCCACCCAAAATAATCTCTCCTAGTATTACCCTAGTGAATGGACATCACTAGTAAGGCGCGGTTTAAATCTCCCGGGGTTCGTGGGGCTGAGGGAACGAGGAAAACAGAAAGGGTGTGGAGATTGGTGAGAGGGAGAGCCAATGATGCGCCAGGCTCCCCGTGAGGCGGAGCTTACCCCGCAGCCTGCCTAACGCTGGTGGGCCAAACACTATCCTGCTCTGGCTATGGGGCGGGGCAAGTCTTACCATTT**GGTCTCGT**AGCACACGCCCCCAGGTGATCTGCGAGCCCAACGATAGGCCATGAGGCCCTGGGCGCGCGCGCGGAGATTGGCTGACGCAGCTTAGAGGCGAGCGGGGATAGGTTACTGGGCTGGCGG |
